# Chronic Paroxetine Treatment Prevents the Emergence of Abnormal Electroencephalogram Oscillations in Huntington’s Disease Mice

**DOI:** 10.1007/s13311-017-0546-7

**Published:** 2017-06-26

**Authors:** Sandor Kantor, Janos Varga, Shreya Kulkarni, A. Jennifer Morton

**Affiliations:** Department of Physiology, Development and Neuroscience, University of Cambridge, Cambridge, UK

**Keywords:** SSRI, Quantitative EEG, Gamma power, Theta oscillation, Biomarker

## Abstract

Disturbance of rapid eye movement (REM) sleep appears early in both patients with Huntington’s disease (HD) and mouse models of HD. Selective serotonin reuptake inhibitors are widely prescribed for patients with HD, and are also known to suppress REM sleep in healthy subjects. To test whether selective serotonin reuptake inhibitors can correct abnormal REM sleep and sleep-dependent brain oscillations in HD mice, we treated wild-type and symptomatic R6/2 mice acutely with vehicle and paroxetine (5, 10, and 20 mg/kg). In addition, we treated a group of R6/2 mice chronically with vehicle or paroxetine (20 mg/kg/day) for 8 weeks, with treatment starting before the onset of overt motor symptoms. During and after treatment, we recorded electroencephalogram/electromyogram from the mice. We found that both acute and chronic paroxetine treatment normalized REM sleep in R6/2 mice. However, only chronic paroxetine treatment prevented the emergence of abnormal low-gamma (25–45 Hz) electroencephalogram oscillations in R6/2 mice, an effect that persisted for at least 2 weeks after treatment stopped. Chronic paroxetine treatment also normalized REM sleep theta rhythm in R6/2 mice, but, interestingly, this effect was restricted to the treatment period. By contrast, acute paroxetine treatment slowed REM sleep theta rhythm in WT mice but had no effect on abnormal theta or low-gamma oscillations in R6/2 mice. Our data show that paroxetine treatment, when initiated before the onset of symptoms, corrects both REM sleep disturbances and abnormal brain oscillations, suggesting a possible mechanistic link between early disruption of REM sleep and the subsequent abnormal brain activity in HD mice.

**Electronic supplementary material:**

The online version of this article (doi:10.1007/s13311-017-0546-7) contains supplementary material, which is available to authorized users.

## Introduction

Huntington’s disease (HD) is an incurable neurodegenerative disorder characterized by motor, cognitive, and psychiatric disturbances, including depression [1]. Disrupted sleep and abnormal brain oscillations typically appear before the diagnostic motor signs, and may contribute to the cognitive impairment and depression seen in patients with HD [2–5]. One of the earliest and most consistent sleep findings in HD is the disturbance of rapid eye movement (REM) sleep. Patients with HD have longer REM sleep latencies, decreased REM sleep amount, and reduced electroencephalogram (EEG) theta oscillations during REM sleep [2, 4–8]. Similar to HD patients, mouse models of HD also have REM sleep disturbances early in the disease process [9–15]. In particular, we have shown previously that R6/2 mice exhibit a progressive increase in REM sleep amount during their active dark period, as well as a slowing of REM sleep EEG theta rhythm [9, 10]. R6/2 mice also develop abnormal low-gamma (25–45 Hz) oscillations in their sleep EEG, including REM sleep [9, 10], reflecting those changes seen in patients with early HD [2]. Similar sleep and EEG abnormalities have been found by other groups in both R6/2 [11, 16, 17] and R6/1 transgenic mice [12, 14, 15], as well as in the Q175 knock-in mouse model of HD [13, 18, 19]. REM sleep plays an important role in stabilizing and refining neuronal circuits in the developing brain [20–22]. Thus, an early disruption of REM sleep may lead to abnormal brain activity that is reflected in the disrupted sleep architecture and abnormal brain oscillations, which are seen not only in patients with HD, but also in mouse models of the disease.

In patients with HD, symptoms of depression are commonly treated with antidepressants, such as the tricyclic antidepressant amitriptyline or the selective serotonin reuptake inhibitor (SSRI) paroxetine [23, 24]. Most antidepressants (including amitriptyline and paroxetine) are known to alter sleep architecture, primarily by suppressing REM sleep [25]. Previously, we have shown that a single dose of amitriptyline normalizes REM sleep and supresses the abnormal EEG oscillations in R6/2 mice [10]. To test whether an antidepressant from a different class, but with similar REM-suppressing effect in healthy subjects, can normalize sleep-dependent brain oscillations in HD mice, we treated wild-type (WT) and R6/2 mice acutely with paroxetine and then monitored the changes in their sleep and EEG. Since changes in sleep and brain oscillatory activity precede most other symptoms in HD, we also treated a group of R6/2 mice chronically with paroxetine from an age that precedes the onset of overt motor symptoms by several weeks (presymptomatic stage). We wanted to see whether the suppression of REM sleep by paroxetine at an early presymptomatic stage of the disease can prevent the development of EEG abnormalities in HD mice. We found that chronic, but not acute, treatment with paroxetine not only normalized REM sleep, but also prevented the development of abnormal brain oscillations in R6/2 mice. Some of these changes persisted for at least 2 weeks after treatment stopped, suggesting that paroxetine treatment had an ameliorating effect on system changes underlying EEG abnormalities seen in HD mice.

## Methods

### Animals and Housing Conditions

All experiments were conducted under the authority of United Kingdom Animals (Scientific Procedures) Act 1986 and with the approval of University of Cambridge Animal Welfare and Ethical Review Panel, and are in compliance with the ARRIVE guidelines. R6/2 and WT littermate mice were taken from a colony established at the University of Cambridge (CBA × C57/BL6 background). Genotyping and repeat length measurements were performed by Laragen (Los Angeles, CA, USA), as described previously [9]. R6/2 mice had a mean CAG repeat length of 252 ± 3. In the acute drug treatment study, 8 WT and 10 R6/2 male mice were used. Before the end of study, 1 WT and 1 R6/2 mouse lost their EEG/electromyogram (EMG) implants and were euthanized. In addition, 1 R6/2 mouse died of its disease. In the chronic drug treatment experiment, 12 male R6/2 mice were used. One R6/2 mouse lost its EEG/EMG implant before the end of study and was euthanized.

### Surgery and EEG/EMG Recordings

We implanted each mouse with EEG and EMG electrodes under isoflurane anesthesia (1.5–2%) [10]. Briefly, we placed screw electrodes epidurally over the frontal (1.5 mm lateral and 1.0 mm anterior to bregma) and parietal (1.5 mm lateral and 1.0 mm anterior to lambda) cortices for frontoparietal EEG recordings. EMG signals were acquired by a pair of stainless steel spring wires inserted into the neck extensor muscles. At the time of surgery, mice were 9 to 10 weeks of age.

After surgery, mice were housed individually under standard conditions [10]. After a recovery period of 7 to 10 days, we connected the mice to recording cables and left them to acclimatize for an additional 3 to 4 days before recording their EEG/EMG signals. The mice then remained connected to the recording cable throughout the study.

EEG/EMG signals were amplified and filtered (EEG: 0.5–60 Hz; EMG: 10–100 Hz) by head-mounted preamplifiers and amplifiers (8202-DSL and 8206-SL, respectively; Pinnacle Technology, Lawrence, KS, USA), and recorded on a computer (Vital Recorder, Kissei Comtec, Matsumoto, Japan) after analog-to-digital conversion.

### Drug Administration

#### Acute Treatment

We treated WT and R6/2 mice acutely with 3 different doses of paroxetine (5, 10, or 20 mg/kg; Sigma-Aldrich, Gillingham, UK) or vehicle (0.9% saline). To test whether paroxetine can correct the abnormally increased REM sleep seen in R6/2 mice during the active dark period [9, 10], we treated the mice just before the onset of the dark period. Then we recorded their sleep–wake behavior for 24 h. The mice were between 12 and 14 weeks of age at the time of treatment. The mice were given vehicle or paroxetine by intraperitoneal injection in a volume of 10 ml/kg body weight. The different doses of paroxetine and its vehicle were given to the mice in a crossover design and in a randomized order, with 3 to 4 days between the treatments. The doses were chosen based on the literature [26–28] and our pilot experiments (data not shown).

#### Chronic Treatment

We treated R6/2 mice daily for 8 weeks with either 20 mg/kg paroxetine (*n* = 6), or vehicle (*n* = 6), with treatment starting at 6 weeks of age. At the age of 9 to 10 weeks, we implanted the mice with EEG/EMG electrodes. The mice were not treated with paroxetine on the day of surgery. We recorded sleep–wake behavior in the mice on several different occasions during treatment, and on 2 occasions after treatment had stopped. On EEG/EMG recording days, the mice received the treatment just before the onset of the dark period, and their sleep–wake behavior was recorded from the beginning of dark period for 24 h, as it was in the acute study. This allowed us to compare directly the effect of acute and chronic treatments on sleep and EEG parameters in R6/2 mice. On the days when no recordings were made during the chronic study, the mice were treated at random times between 8 a.m. and 4 p.m. during the day, to avoid any potential zeitgeber effect of treatments.

Successful EEG/EMG recordings with all four treatments were achieved in 6/8 WT mice in the acute drug study. Of the other 2 WT mice, 1 WT mouse received all treatments apart from the 5 mg/kg dose of paroxetine and 1 WT mouse received vehicle and the 20 mg/kg doses of paroxetine. Seven of 10 R6/2 mice received all the treatments in the acute drug experiment. Of the other 3 R6/2 mice, 2 received vehicle and all doses of paroxetine, apart from either the 5 mg/kg (1 mouse) or the 20 mg/kg (1 mouse) doses of the drug. One R6/2 mouse received vehicle and the 20 mg/kg dose of paroxetine.

In the chronic drug treatment experiment, successful EEG/EMG recordings were made in all 6 paroxetine-treated and 5/6 vehicle-treated R6/2 mice at all time points. One vehicle-treated R6/2 mouse had all the EEG/EMG recordings made from it, except the final recording that should have been done 2 weeks after drug treatment stopped.

### Data Analysis and Statistics

All signals were digitized at 256 Hz, digitally filtered (EEG: 0.5–60 Hz; EMG: 10–60 Hz), and semi-automatically scored as wake, non-REM (NREM) sleep, or REM sleep in 10-s epochs using SleepSign (Kissei Comtec, Matsumoto, Japan). Experienced scorers, blinded to treatment and genotype, visually inspected these preliminary scorings and made corrections when appropriate. We then measured the duration of bouts, counted the number of bouts, and calculated the time spent in each behavioral state during both dark and light periods. We also analyzed the probability of transitioning into REM sleep as a function of NREM sleep bout length, as described previously [9].

To reveal the changes in the frequency content of the recorded signal, we performed a power spectral analysis of the EEG after the treatments. EEG power spectra were computed for artifact-free 2-s epochs by fast Fourier transformation, as described previously [10]. The values of consecutive 2-s EEG epochs in wake, NREM, and REM sleep were averaged over 12 h after the treatments. Data are presented at 0.5-Hz resolution or in 1-Hz bins, where the bins were marked by their upper limits. The spectral values in each frequency bin were normalized to the total power of the studied EEG spectrum (0.5–49 Hz) over 24 h and log-transformed for graphical presentation, or were normalized to the mean power spectral values of vehicle-treated mice of the same genotype (100%). To reveal the changes in specific frequency bands after the treatments, in addition to analyzing the entire EEG spectrum, we also compared the discrete changes in the theta (4–10 Hz) and low-gamma (25–45 Hz) bands of the EEG.

To compare statistically the vigilance state parameters and normalized EEG data, we used multivariate analysis of variance with repeated measures and unpaired *t*-tests (Statistica 13; Statsoft, Tulsa, OK, USA). The results were considered statistically significant at *p* < 0.05. All results are expressed as means ± SEM.

## Results

### Paroxetine Suppresses REM Sleep and Consolidates NREM Sleep

**Fig. 1:**
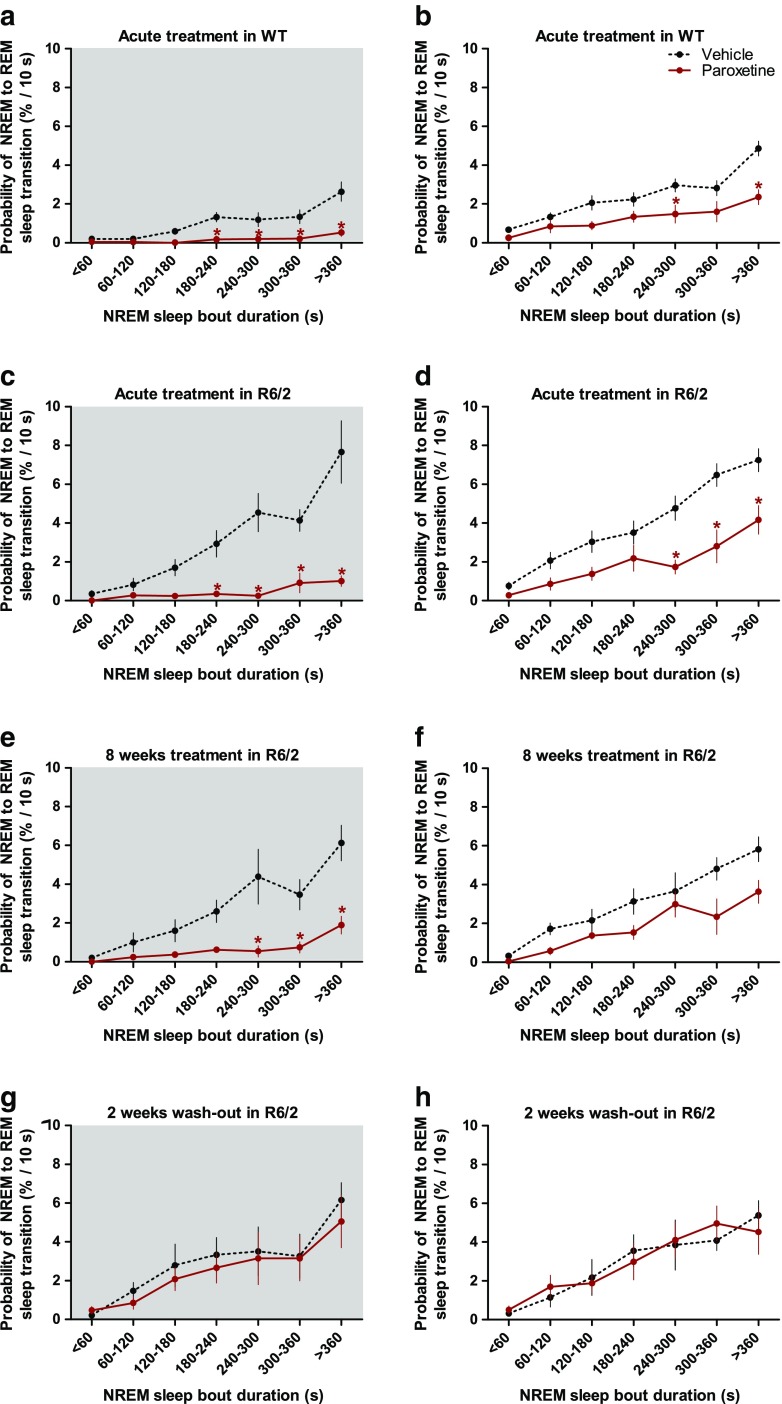
Paroxetine decreases the probability of entering into rapid eye movement (REM) sleep from a non-REM (NREM) sleep episode. The probability of transitioning into REM sleep as a function of NREM sleep bout length as shown in (a, b) wild-type (WT) and (c–h) R6/2 mice during dark and light periods after (a–d) acute or (e, f) an 8-week-long chronic treatment with vehicle (dashed line) or paroxetine (20 mg/kg i.p.; solid line), as well as (g, h) after a washout period of 2 weeks postchronic treatments. The absolute probability of transitioning from NREM into REM sleep for each 10-s epoch of NREM sleep was calculated and then the weighted average probability for bins of increasing duration (<60, 60–120, 120–180, 180–240, 240–300, 300–360, and > 360 s) was presented as group mean ± SEM. The dark period is shown as shaded area. *p < 0.05 *vs* vehicle treatment (Bonferroni post-test)

Acute treatment with paroxetine dose-dependently decreased the amount of REM sleep in both WT and R6/2 mice [drug effect: F_(3,33)_ = 20.26; *p* < 0.01]. During the first 12 h, the amount of REM sleep was decreased by > 60% in both WT and R6/2 mice after the highest dose (20 mg/kg i.p.) of paroxetine (Table 1). The REM sleep-suppressing effect of paroxetine was restricted to the first 12 h in WT mice but extended into the second 12 h in R6/2 mice [drug × dark/light period interaction: F_(3,33)_ = 4.01; *p* < 0.05 (Table 1)]. Paroxetine suppressed REM sleep by reducing its propensity in both genotypes, as shown by a reduction in the number of REM sleep bouts [drug effect: F_(3,33)_ = 21.79; *p* < 0.01 (Table 1)]. The reduction in REM sleep propensity is also shown by the decreased probability of transitioning from NREM sleep into REM sleep in paroxetine-treated mice, irrespective of genotype or dark/light period [drug effect: F_(1,14)_ = 37.36; *p* < 0.01 (Fig. 1a–d)]. In WT mice, paroxetine also improved the maintenance of REM sleep, as indicated by longer REM sleep bouts after paroxetine treatment than after vehicle treatment [drug effect: F_(3,30)_ = 4.99; *p* < 0.01 (Table 1)]. However, this effect of paroxetine depended on the dark/light period and could be seen during the light period only [drug × dark/light period interaction: F_(3,30)_ = 3.40; *p* < 0.05 (Table 1)].

**Table 1:** Vigilance state parameters in wild-type (WT) and R6/2 mice after vehicle or paroxetine treatment

Genotype	WT				R6/2			
Dose	Vehicle (*n* = 8)	5 mg/kg (*n* = 6)	10 mg/kg (*n* = 7)	20 mg/kg (*n* = 8)	Vehicle (*n* = 10)	5 mg/kg (*n* = 8)	10 mg/kg (*n* = 9)	20 mg/kg (*n* = 9)
Wake								
Total time (min)								
Dark period	350.0 ± 20.5	307.1 ± 16.8	289.5 ± 17.5*	298.3 ± 14.5*	305.7 ± 18.3	297.7 ± 17.6	275.5 ± 12.0	271.4 ± 10.5
Light period	184.3 ± 7.5	187.1 ± 12.1	179.6 ± 5.9	182.4 ± 10.0	212.5 ± 8.4	209.2 ± 8.5	205.9 ± 10.1	217.8 ± 8.8
Mean duration (min)								
Dark period	5.2 ± 0.9	4.5 ± 0.4	4.2 ± 0.4	4.6 ± 0.6	4.0 ± 0.4	4.9 ± 0.3	4.9 ± 0.3	4.9 ± 0.7
Light period	2.7 ± 0.4	2.7 ± 0.5	2.4 ± 0.3	2.1 ± 0.1	2.6 ± 0.2	3.0 ± 0.2	3.0 ± 0.3	3.1 ± 0.3
Number of bouts								
Dark period	78.6 ± 11.1	70.5 ± 3.4	71.9 ± 6.9	70.8 ± 6.1	79.8 ± 5.0	61.4 ± 4.6	56.8 ± 2.6	62.6 ± 7.7
Light period	73.3 ± 6.8	74.5 ± 6.3	80.1 ± 6.7	90.0 ± 5.0	82.2 ± 4.8	70.1 ± 3.4	72.2 ± 6.0	72.7 ± 6.3
NREM sleep								
Total time (min)								
Dark period	338.1 ± 21.6	389.7 ± 18.9	417.3 ± 19.5^*^	411.7 ± 16.0*	351.8 ± 17.5	377.9 ± 16.7	410.6 ± 13.2*	426.7 ± 10.8^*^
Light period	456.3 ± 8.4	454.6 ± 11.5	471.6 ± 5.7	472.5 ± 13.2	408.6 ± 7.8	417.2 ± 8.7	428.0 ± 8.3	421.6 ± 9.4
Mean duration (min)								
Dark period	3.9 ± 0.3	5.3 ± 0.2	6.0 ± 0.7	6.0 ± 0.6*	3.3 ± 0.2	5.5 ± 0.4*	6.5 ± 0.3*	7.0 ± 0.7*
Light period	3.9 ± 0.2	4.2 ± 0.3	4.9 ± 0.4	4.7 ± 0.3	3.0 ± 0.2	3.8 ± 0.2	4.5 ± 0.3	4.9 ± 0.5*
Number of bouts								
Dark period	89.9 ± 10.6	74.8 ± 4.9	74.1 ± 6.8	71.5 ± 5.9*	108.7 ± 6.8	70.8 ± 4.9*	63.7 ± 2.1*	67.6 ± 8.0*
Light period	119.0 ± 8.5	111.0 ± 9.3	98.9 ± 7.5	103.4 ± 4.8*	137.1 ± 7.0	112.1 ± 5.0	98.9 ± 7.5*	91.9 ± 8.4*
REM sleep								
Total time (min)								
Dark period	31.9 ± 5.2	23.2 ± 2.9	13.2 ± 3.0	10.0 ± 2.9*	62.5 ± 3.2	44.4 ± 3.5*	33.9 ± 2.8*	21.9 ± 4.5*
Light period	79.5 ± 2.9	78.3 ± 3.3	68.8 ± 2.2	65.0 ± 5.2	98.9 ± 3.3	93.7 ± 3.1	86.1 ± 3.8	80.7 ± 3.4*
Mean duration (min)								
Dark period	1.3 ± 0.1	1.4 ± 0.1	1.7 ± 0.2	1.7 ± 0.2	1.3 ± 0.1	1.5 ± 0.1	1.6 ± 0.1	1.5 ± 0.2
Light period	1.2 ± 0.1	1.3 ± 0.1	1.6 ± 0.2	1.8 ± 0.2*	1.2 ± 0.1	1.4 ± 0.1	1.5 ± 0.1	1.7 ± 0.1
Number of bouts								
Dark period	23.4 ± 3.5	16.7 ± 1.6	8.3 ± 2.5	6.4 ± 2.1	50.0 ± 4.5	30.0 ± 3.5*	21.8 ± 1.2*	15.7 ± 3.9*
Light period	68.3 ± 3.2	63.2 ± 4.5	46.0 ± 5.7*	39.9 ± 6.4*	82.1 ± 6.5	69.5 ± 5.3	56.6 ± 4.0*	51.4 ± 6.2*

Total time spent in each state, mean duration, and number of bouts during the dark (active) and light (passive) periods. Data are shown as mean ± SEM. * p < 0.05 *vs* vehicle treatment of the same genotype (Bonferroni post-test)

NREM = non-rapid eye movement; REM = rapid eye movement

In addition to REM sleep suppression, paroxetine exerted a dose-dependent hypnotic effect in both WT and R6/2 mice [drug effect: F_(3,33)_ = 9.12; *p* < 0.01 (Table 1)]. This hypnotic effect of paroxetine, shown by an increase in the amount of NREM sleep, was restricted to the first 12 h of treatment in both WT and R6/2 mice [drug × dark/light period interaction: F_(3,33)_ = 8.93; *p* < 0.01 (Table 1)]. Paroxetine also consolidated NREM sleep by increasing the mean duration and reducing the number of NREM sleep bouts in both genotypes [drug effects: F_(3,33)_ = 23.62 (*p* < 0.01) and F_(3,33)_ = 17.36 (*p* < 0.01), respectively (Table 1)]. The paroxetine-induced changes in NREM sleep bout duration were restricted to the dark period in WT mice but continued into the light period in R6/2 mice [drug × dark/light period interaction: F_(3,33)_ = 5.57; *p* < 0.01 (Table 1)]. In WT mice, paroxetine also decreased the amount of wakefulness, but this effect was limited to the first 12 h after drug administration [drug × dark/light period interaction: F_(3,33)_ = 4.55; *p* < 0.01 (Table 1)].

### Paroxetine Slows Down REM Sleep Theta Rhythm

**Fig. 2:**
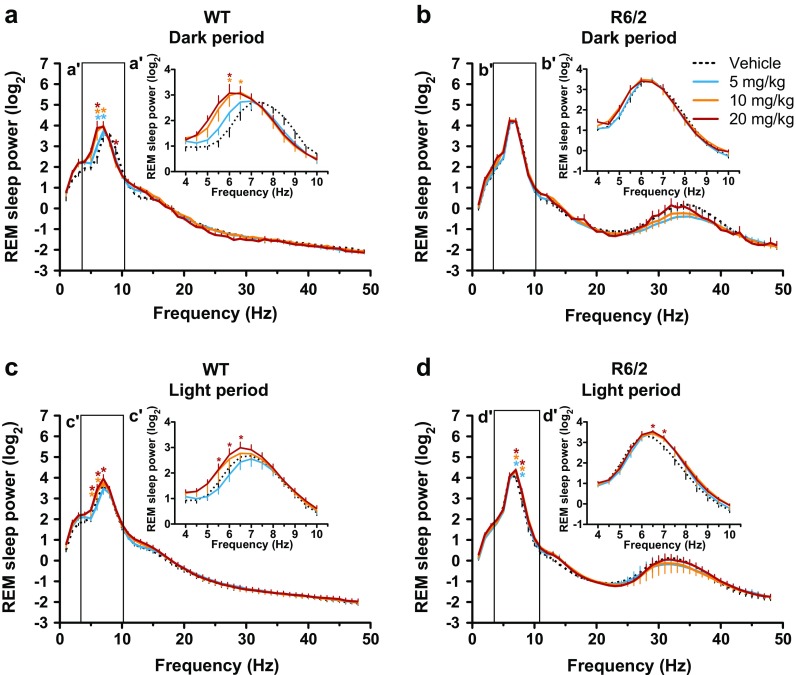
Paroxetine reduces the frequency of rapid eye movement (REM) sleep theta oscillations in wild-type (WT) mice close to the one seen in R6/2 mice. Changes in relative power values of the electroencephalogram (EEG) spectra during REM sleep as shown in (a, c) WT and (b, d) R6/2 mice during the (a, b) dark and (c, d) light period after vehicle or paroxetine (5, 10, and 20 mg/kg i.p.) treatment. Enlarged images of relative EEG power values in the theta band (4–10 Hz) outlined by the box are shown in the insets. Data are shown as mean ± SEM in (a–d) 1-Hz bins or (a'–d') at 0.5-Hz resolution. *p < 0.05 *vs* vehicle treatment (Bonferroni post-test)

**Fig. 3:**
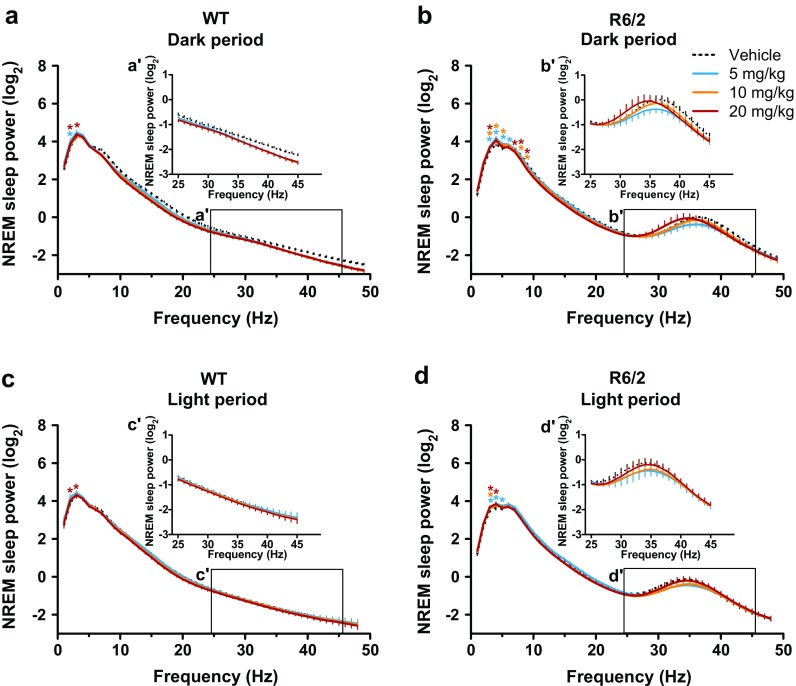
Acute paroxetine treatment had no effect on the abnormal low-gamma electroencephalogram (EEG) oscillations in R6/2 mice. Changes in relative power values of EEG spectra during non-rapid eye movement (NREM) sleep as shown in (a, c) wild-type (WT) and (b, d) R6/2 mice during the (a, b) dark and (c, d) light period after vehicle or paroxetine (5, 10, and 20 mg/kg i.p.) treatment. Enlarged images of relative EEG power values in the low-gamma band (25–45 Hz) outlined by the box are shown in the insets. Data are shown as mean ± SEM in 1-Hz bins. *p < 0.05 *vs* vehicle treatment (Bonferroni post-test)

In WT mice, the acute effect of paroxetine treatment on REM sleep EEG spectra was restricted to the peak frequencies of the theta band (5–7 Hz) during both dark and light periods [drug × frequency interactions: F_(144,576)_ = 2.42 (*p* < 0.01) and F_(144,720)_ = 2.47 (*p* < 0.01), respectively (Fig. 2a, c)]. Specifically, paroxetine shifted the peak frequency of REM sleep theta oscillation from 7 Hz to 6 Hz in WT mice during the dark period [drug × frequency interaction: F_(36,144)_ = 2.47; *p* < 0.01 (Fig. 2a')]. Interestingly, this is similar to the pathological theta peak frequency seen in symptomatic R6/2 mice (6–6.5 Hz in vehicle-treated mice; Fig. 2b'). The paroxetine-induced slow down of REM sleep theta rhythm persisted throughout the light period in WT mice [drug × frequency interaction: F_(36,180)_ = 1.85; *p* < 0.01 (Fig. 2c')]. In R6/2 mice, however, paroxetine had no further effect on the already-slowed EEG theta rhythm seen during the dark period (Fig. 2b, b'). Interestingly, paroxetine improved REM sleep EEG spectra in R6/2 mice during the second 12 h [drug effect: F_(3,18)_ = 6.09; *p* < 0.01 (Fig. 2d)]. The rhythm of REM sleep theta oscillation became slightly faster during the light period in R6/2 mice after paroxetine treatment than after vehicle treatment [6.5 Hz *vs* 6.0 Hz; drug × frequency interaction: F_(36,216)_ = 1.79; *p* < 0.01 (Fig. 2d')]. Furthermore, paroxetine increased the power of slow oscillations (2–6 Hz) in the NREM sleep EEG of WT mice during both dark and light periods [drug × frequency interactions: F_(144,720)_ = 1.53 (*p* < 0.01) and F_(144,720)_ = 1.27 (*p* < 0.05), respectively (Fig. 3a, c)]. A similar increase in EEG slow oscillations was seen in R6/2 mice during NREM sleep after acute paroxetine treatment during both dark and light periods [drug × frequency interactions: F_(144,864)_ = 4.03 (*p* < 0.01) and F_(144,864)_ = 2.88 (*p* < 0.01), respectively (Fig. 3b, d)]. Acute paroxetine treatment, however, had no effect on the abnormal low-gamma EEG oscillations seen in R6/2 mice (Fig. 3b', d').

### Chronic Treatment With Paroxetine Normalizes REM Sleep in HD Mice

**Fig. 4:**
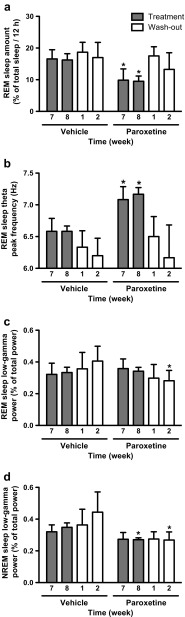
Chronic paroxetine treatment normalizes rapid eye movement (REM) sleep, as well as theta and low-gamma electroencephalogram (EEG) oscillations in R6/2 mice. Changes in (a) the amount of REM sleep, (b) REM sleep theta peak frequency, (c) REM sleep low-gamma power (25–45 Hz), and (d) non-REM (NREM) sleep low-gamma power is shown in vehicle- and paroxetine (20 mg/kg/day)-treated R6/2 mice during the dark period after 7 and 8 weeks of treatment (closed bars), as well as 1 and 2 weeks after treatment stopped (“washout”, open bars). Theta peak frequency was defined as the frequency value (0.5-Hz resolution) within the theta range (4–10 Hz) with the highest EEG power value. Data are shown as mean ± SEM. *p < 0.05 *vs* vehicle treatment at the corresponding time (unpaired *t*-test)

In both acute and chronic studies, vehicle-treated R6/2 mice had an increased propensity for REM sleep during the dark period compared to that of WT mice receiving acute vehicle treatment. This is shown by the doubled amount of REM sleep, increased number of REM sleep bouts, and an increased probability of entering REM sleep from NREM sleep (Tables 1 and 2; Fig. 1). By contrast, the propensity for REM sleep was normalized in R6/2 mice treated chronically with paroxetine (20 mg/kg/day i.p.). This is shown by the decreased amount of REM sleep during the dark period seen in R6/2 mice that was significant after 7 weeks of paroxetine treatment [drug effect: F_(1,9)_ = 5.30; *p* < 0.05 (see Fig. 4a)]. Eight weeks of treatment with paroxetine resulted in a 42% decrease in REM sleep amount and a 60% decrease in the number of REM sleep bouts in R6/2 mice during the dark period [drug × dark/light period interactions: F_(1,10)_ = 8.47 (*p* < 0.05) and F_(1,10)_ = 8.50 (*p* < 0.05), respectively (Table 2)]. Furthermore, the probability of entering REM sleep in the dark period gradually increased across the duration of NREM sleep in vehicle-treated R6/2 mice. By contrast, R6/2 mice treated with paroxetine had a much lower probability of entering REM sleep at any time during a NREM sleep episode if that was longer than 240 s [drug × NREM sleep bout duration interaction: F_(6,60)_ = 5.69; *p* < 0.01 (Fig. 1e)]. Interestingly, chronic treatment with paroxetine corrected REM sleep abnormalities in R6/2 mice during the dark period but had little effect on REM sleep parameters during the light period (Table 2; Fig. 1f). Only the mean duration of REM sleep bouts changed during both dark and light periods in paroxetine-treated R6/2 mice. REM sleep bouts became longer [drug effect: F_(1,10)_ = 19.54; *p* < 0.01 (Table 2)], suggesting that R6/2 mice had a better consolidated REM sleep after chronic paroxetine treatment than after vehicle treatment. Paroxetine did not change any other sleep–wake parameters measured (Table 2). Furthermore, none of the changes in REM sleep parameters induced by paroxetine persisted after treatment stopped (Table 2; Fig. 1g, h, and Fig. 4a).

**Table 2:** Vigilance state parameters in R6/2 mice after chronic treatment with vehicle or paroxetine

Recording	8 weeks of treatment		2 weeks of washout	
Treatment	Vehicle (*n* = 6)	Paroxetine (*n* = 5)	Vehicle (*n* = 6)	Paroxetine (*n* = 6)
Wake				
Total time (min)				
Dark period	277.9 ± 7.1	271.0 ± 25.2	312.1 ± 24.5	308.8 ± 22.5
Light period	227.3 ± 15.1	219.9 ± 16.3	234.2 ± 49.6	297.6 ± 38.5
Mean duration (min)				
Dark period	3.3 ± 0.4	3.4 ± 0.7	4.2 ± 0.6	3.9 ± 0.5
Light period	2.9 ± 0.3	3.0 ± 0.4	3.0 ± 0.6	3.3 ± 0.5
Number of bouts				
Dark period	90.5 ± 10.5	91.5 ± 14.3	79.2 ± 7.8	82.8 ± 9.1
Light period	83.0 ± 9.8	77.8 ± 6.4	77.8 ± 7.0	94.0 ± 10.0
NREM sleep				
Total time (min)				
Dark period	369.4 ± 11.3	407.1 ± 26.1	336.3 ± 19.9	354.7 ± 25.2
Light period	397.9 ± 13.1	409.7 ± 13.7	396.5 ± 40.1	351.9 ± 30.1
Mean duration (min)				
Dark period	3.3 ± 0.2	4.4 ± 0.4	3.3 ± 0.2	3.7 ± 0.6
Light period	3.4 ± 0.3	4.4 ± 0.3	3.6 ± 0.3	3.1 ± 0.4
Number of bouts				
Dark period	114.2 ± 6.9	97.2 ± 13.9	105.2 ± 10.1	106.0 ± 12.6
Light period	118.8 ± 8.3	95.2 ± 9.7	110.0 ± 7.8	118.8 ± 13.5
REM sleep				
Total time (min)				
Dark period	72.7 ± 8.9	41.9 ± 6.5*	71.6 ± 8.9	56.6 ± 8.1
Light period	94.8 ± 6.7	90.4 ± 3.1	89.2 ± 12.8	70.6 ± 13.8
Mean duration (min)				
Dark period	1.5 ± 0.0	2.3 ± 0.2^*^	1.5 ± 0.2	1.4 ± 0.1
Light period	1.5 ± 0.1	2.1 ± 0.2^*^	1.5 ± 0.1	1.4 ± 0.1
Number of bouts				
Dark period	48.0 ± 5.8	19.0 ± 3.6*	50.0 ± 7.8	42.5 ± 7.5
Light period	63.2 ± 4.8	44.8 ± 5.4	60.6 ± 9.4	49.5 ± 9.1

Total time spent in each state, mean duration, and number of bouts during the dark (active) and light (passive) periods after 8 weeks of treatment with vehicle or paroxetine (20 mg/kg/day i.p.) or after a 2-week washout period. Data are shown as mean ± SEM. * p < 0.05 *vs* vehicle-treated group of the same recording day (Bonferroni post-test)

### Chronic Paroxetine Treatment Prevents EEG Abnormalities in HD Mice

**Fig. 5:**
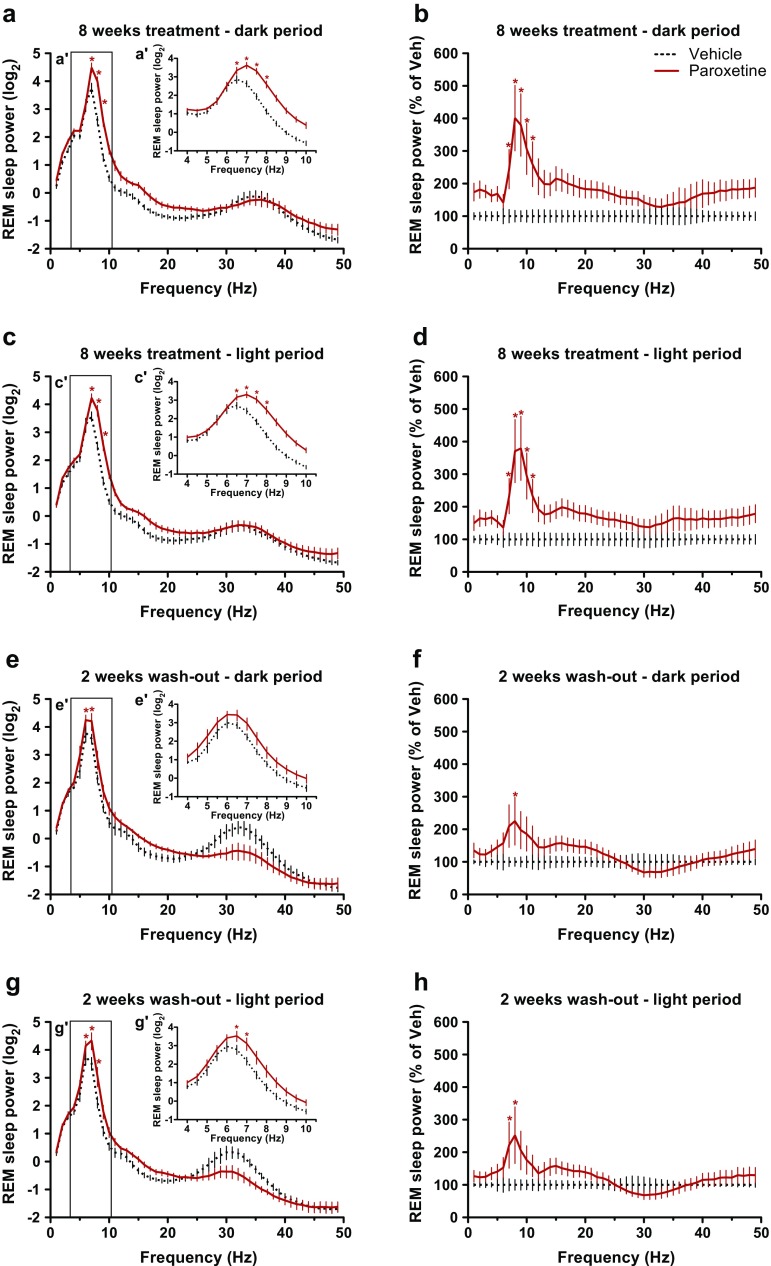
Chronic treatment with paroxetine prevents the slowdown of rapid eye movement (REM) sleep theta rhythm in R6/2 mice. Changes in relative power values of electroencephalogram (EEG) spectra during REM sleep as shown in R6/2 mice during the (a, b, e, f) dark and (c, d, g, h) light period after (a–d) an 8-week-long chronic vehicle (dashed line) or paroxetine (20 mg/kg/day i.p.; solid line) treatment, as well as (e–h) after a washout period of 2 weeks after chronic treatment. The spectral values were normalized to (a, c, e, g) the total power of the studied EEG spectrum or to (b, d, f, h) the mean power spectral values of vehicle-treated mice. Enlarged images of relative EEG power values in the theta band (4–10 Hz) outlined by the box are shown in the insets. Data are shown as mean ± SEM in (a, c, e, g) 1-Hz bins or at (a', c', e', g') 0.5 Hz resolution. *p < 0.05 *vs* vehicle treatment (Bonferroni post-test)

At 14 weeks of age, the peak frequency of REM sleep theta oscillations was 6.5 Hz in vehicle-treated R6/2 mice during both dark and light periods (Fig. 5a', c'). That is 0.5 to 1 Hz slower than the peak frequency of the REM sleep theta oscillation (7–7.5 Hz) seen in WT mice after acute vehicle treatment (Fig. 2a', c'). Paroxetine normalized REM sleep EEG spectra in chronically treated R6/2 mice during both dark and light periods [drug effects: F_(1,10)_ = 23.36 (*p* < 0.01) and F_(1,10)_ = 17.68 (*p* < 0.01), respectively (Fig. 5a, c)]. In R6/2 mice, REM sleep theta rhythm became more robust (with an increase in power between 7 and 11 Hz) after paroxetine treatment than after vehicle treatment during both dark and light periods [drug effects: F_(1,10)_ = 5.50 (*p* < 0.05) and F_(1,10)_ = 5.66 (*p* < 0.05), respectively (Fig. 5b, d)]. REM sleep theta rhythm also became faster (with a peak at 7 Hz) in R6/2 mice after paroxetine treatment than after vehicle treatment during both the dark and light periods [drug × frequency interactions: F_(12,120)_ = 8.80 (*p* < 0.01) and F_(12,120)_ = 7.47 (*p* < 0.01), respectively (Fig. 5a', c')]. REM sleep theta rhythm was already faster in paroxetine-treated than in vehicle-treated R6/2 mice after 7 weeks of treatment [time × drug interaction: F_(3,27)_ = 3.70; *p* < 0.05 (Fig. 4b)]. Two weeks after the treatment was finished, the drug-induced differences in theta rhythm between the groups were reduced (Fig. 4b and Fig. 5e–h). Specifically, the rhythm of REM sleep theta was still slightly faster in paroxetine-treated R6/2 mice than in vehicle-treated mice during the light period [6.5 *vs* 6 Hz; drug × frequency interaction: F_(12,108)_ = 1.90; *p* < 0.05 (Fig. 5g')] but not during the dark period 2 weeks after treatment stopped (Fig. 5e' and Fig. 4b).

**Fig. 6:**
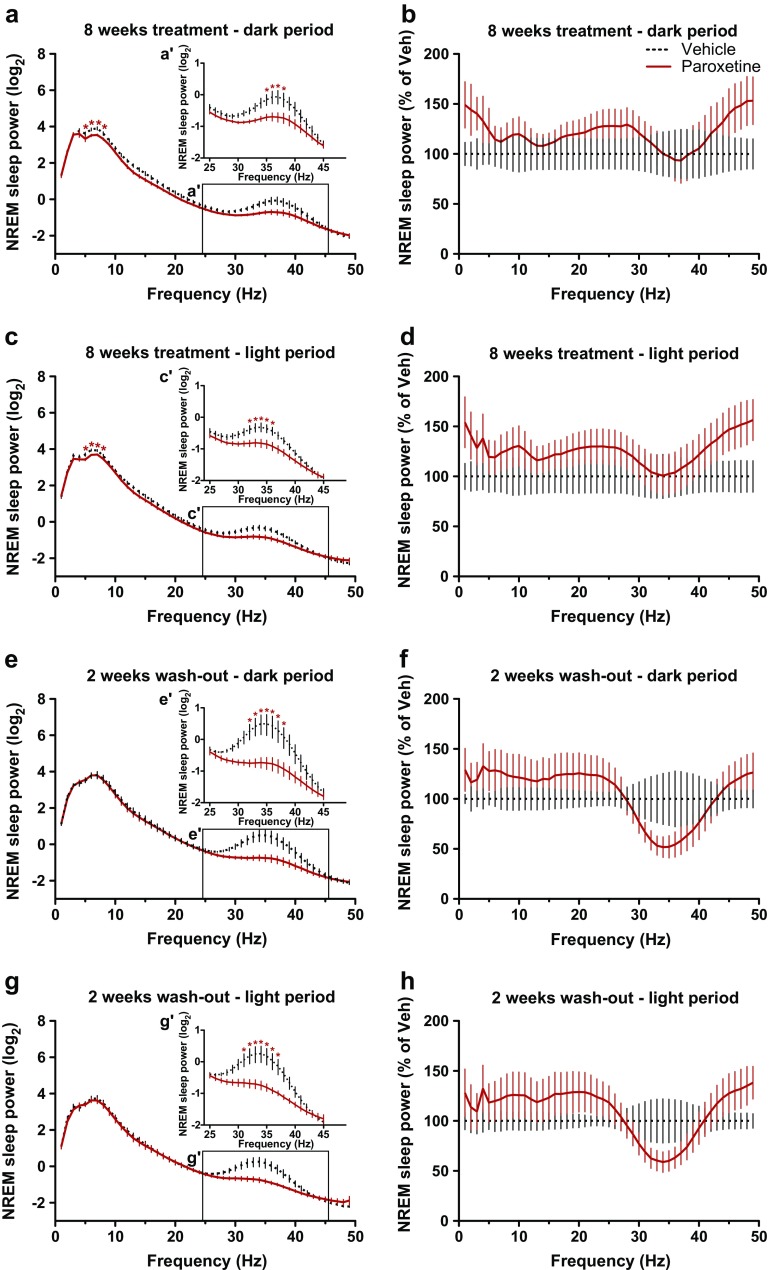
Chronic paroxetine treatment prevents abnormal low gamma electroencephalogram (EEG) oscillation in R6/2 mice. Changes in relative power values of EEG spectra during non-rapid eye movement (NREM) sleep are shown in R6/2 mice during the dark (a, b, e, f) and light (c, d,g, h) period after an 8-week-long vehicle (dashed line) or paroxetine (20 mg/kg/day i.p.; solid line) treatment (a–d), as well as after a washout period after the chronic treatment ended of 2 weeks duration (e–h). The spectral values were normalized to the total power (a, c, e, g) of the studied EEG spectrum or to the mean power (b, d, f, h) spectral values of vehicle treated mice. Enlarged images of relative EEG power values in the low-gamma band (25–45Hz) outlined by the box are shown in the insets. Data are shown as mean ± SEM in 1-Hz bins. *p < 0.05 vs vehicle treatment (Bonferroni post-hoc test).

As the disease progressed, an abnormal low-gamma EEG activity emerged in vehicle-treated but not in paroxetine-treated R6/2 mice during both REM and NREM sleep [time × drug interaction: F_(3,27)_ = 9.58 (*p* < 0.01); drug effect: F_(1,9)_ = 7.78 (*p* < 0.05), respectively (Fig. 4c, d)]. After 8 weeks of paroxetine treatment, R6/2 mice had significantly fewer abnormal low-gamma oscillations in their NREM sleep EEG than vehicle-treated mice during both dark and light periods [drug × frequency interactions: F_(20,200)_ = 2.72 (*p* < 0.01) and F_(20,200)_ = 4.01 (*p* < 0.01), respectively (Fig. 6a', c')]. The suppression of low-gamma EEG activity during NREM sleep persisted for at least 2 weeks after treatment stopped in R6/2 mice [time × drug interaction: F_(3,27)_ = 6.10; *p* < 0.01 (Fig. 4d)]. Specifically, as the disease progressed, vehicle-treated R6/2 mice developed an abnormally increased low-gamma EEG activity during NREM sleep (with a peak frequency at 33–35 Hz) during both dark and light periods that was 2-fold higher than the low-gamma activity seen in paroxetine-treated R6/2 mice [drug × frequency interactions: F_(20,180)_ = 4.76 (*p* < 0.01) and F_(20,180)_ = 6.50 (*p* < 0.01), respectively (Fig. 6e', g')]. Chronic paroxetine treatment also prevented the development of abnormal low-gamma EEG oscillations in R6/2 mice during REM sleep, an effect that persisted for at least 2 weeks after treatment stopped, during both dark and light periods [drug × frequency interactions: F_(20,180)_ = 2.60 (*p* < 0.01) and F_(20,180)_ = 2.55 (*p* < 0.01), respectively (Fig. 5e, g)].

## Discussion

We show that chronic treatment with paroxetine, when started at a presymptomatic stage of disease, prevented sleep and EEG abnormalities in the R6/2 mouse model of HD. Control R6/2 mice, treated with vehicle, exhibited an abnormally increased REM sleep amount (particularly during the dark, when they are normally active), slowed REM sleep theta rhythm, and abnormal low-gamma oscillations in their sleep EEG. None of these abnormalities could be seen in R6/2 mice treated chronically with paroxetine, during either dark or light period. Two weeks after treatment stopped, the beneficial effect of paroxetine on REM sleep and REM sleep theta rhythm had largely disappeared. On the one hand, suppression of abnormal EEG gamma activity persisted in the R6/2 mice for at least 2 weeks after the last paroxetine treatment. On the other hand, acute treatment with paroxetine normalized the abnormal increase in REM sleep seen during the active period and consolidated NREM sleep but had no effect on abnormal theta or gamma oscillations in R6/2 mice. In WT mice, acute paroxetine treatment slowed REM sleep theta rhythm to the level seen in symptomatic R6/2 mice, suggesting that 5-hydroxytryptamine (serotonin; 5-HT) neurotransmission is already altered in HD mice. Our data show that paroxetine corrects both disrupted sleep and abnormal brain oscillations in HD mice when prophylactic treatment is initiated before the onset of symptoms.

Patients with HD show disturbed sleep and abnormal brain oscillations early in the disease process, with decreased REM sleep time being one of the most consistent findings [2, 5, 6, 8]. Mice are nocturnal, and HD mice, including the R6/2 mice studied here, already show an abnormal increase in REM sleep amount during the night at presymptomatic stage of the disease [9–13]. REM sleep is generated primarily by brain stem neurons [29, 30]. 5-HT inhibits REM sleep [31], and paroxetine treatment increases the extracellular level of 5-HT [32]. In our study, acute treatment with paroxetine suppressed REM sleep in both WT and R6/2 mice. We previously obtained similar results using the 5-HT/noradrenaline reuptake inhibitor amitriptyline [10]. The REM sleep-suppressing effect of antidepressants is well documented [25]. However, in humans most antidepressants (including paroxetine) suppress REM sleep early in the treatment, and this effect gradually diminishes after repeated administration of the drug [25]. In R6/2 mice, however, paroxetine prevented the abnormal increase in REM sleep amount for at least 8 weeks during treatment. Whether this is a consequence of an enhanced 5-HT neurotransmission or is due to changes in REM sleep-controlling circuits needs further investigation, particularly because drugs may have markedly different effects on the neurologically normal and HD brain.

In patients with HD, EEG theta oscillations are slowed during quiet wakefulness [33], and have decreased power during REM sleep [2, 7]. Symptomatic R6/2 mice in our study had slowed EEG theta oscillations during REM sleep, which is in accord with previous findings in R6/2 [9, 11], R6/1 [14, 15], and Q175 [13] mice. EEG theta oscillations in rodents are thought to have a hippocampal origin [34, 35], and 5-HT neurons play a key role in modulating hippocampal theta oscillations [36]. Here in WT mice, acute treatment with paroxetine reduced the frequency of REM sleep theta rhythm to a level seen in symptomatic R6/2 mice. A similar decrease in reticular-elicited theta frequency has been shown in Sprague–Dawley rats after SSRI fluoxetine treatment [37]. However, paroxetine had no further effect on the already slowed REM sleep theta rhythm in R6/2 mice, suggesting an already altered 5-HT neurotransmission in these mice. Increased signaling through 5-HT_6_ receptors could account for the abnormal theta rhythm seen in HD mice as it has been shown that activation of these receptors decreases hippocampal theta frequency [38]. Although there is no direct evidence of an altered 5-HT signaling in HD, the diminishing of depressive symptoms in patients with late-stage HD [1] and the decrease in 5-HT-dependent behavioral despair in symptomatic R6/2 mice [39] both suggest compensatory changes in brain 5-HT neurotransmission. It should be noted, however, that alterations in neurotransmitter systems other than 5-HT may also underlie the changes we see.

Abnormal EEG gamma oscillations have been reported in several neuropsychiatric disorders, including schizophrenia [40], depression [41], and Alzheimer’s disease [42]. Abnormal EEG gamma oscillations have been found in both patients with early-HD and in presymptomatic R6/2 mice [2, 9–11]. Although the functional significance of abnormal high-frequency EEG oscillations in these neuropsychiatric disorders is unknown, gamma oscillations have attracted a lot of attention in recent years because of the role they play in feature binding [43], object representation [44], and selective attention [45]. Gamma oscillations are generated in the cortex and hippocampus [46, 47]. Since basal ganglia is severely affected in HD, insufficient inhibition of cortical activity by cortical projecting neurons of basal ganglia could account, at least in part, for the abnormal low-gamma EEG oscillations seen in HD [10]. Both cortex and hippocampus express high levels of brain-derived neurotrophic factor (BDNF); a protein that promotes the maintenance and survival of neurons mainly through its tyrosine kinase (Trk) B receptor [48]. BDNF modulates gamma oscillations [49], and BDNF-TrkB signaling is impaired in HD [50]. Chronic (but not acute) treatment with the SSRI paroxetine or fluoxetine, increases BDNF expression and improves disease symptoms in HD mice [27, 28, 51, 52]. Here, chronic treatment with paroxetine suppressed abnormal low-gamma EEG oscillations in R6/2 mice. This is similar to the suppression of EEG gamma activity seen in R6/2 mice after acute amitriptyline treatment [10]. Interestingly, amitriptyline acts on TrkA and TrkB receptors as agonist and has potent neurotrophic activity [53]. Thus, we hypothesize that the correction of abnormal low-gamma EEG oscillations in R6/2 mice by chronic paroxetine treatment may be due, at least in part, to an increase in brain neurotrophic activity. Although instrumental REM sleep deprivation also increases BDNF expression in rodents [54, 55], it needs to be further investigated with regard to whether the paroxetine-induced suppression of REM sleep results in increased neurotrophic activity in the HD mouse brain.

Our data show that paroxetine treatment, when initiated before the onset of symptoms, corrects both REM sleep disturbances and abnormal brain oscillations in HD mice. This suggests a possible mechanistic link between early disruption of REM sleep and abnormal brain oscillations in HD mice. Since abnormal sleep and EEG changes are likely to be correlates of altered brain function in HD, correcting these abnormalities might also be reflected in improvements in HD symptoms other than sleep and sleep-dependent brain oscillations.

## Electronic supplementary material

Below is the link to the electronic supplementary material.

Required Author Forms Disclosure forms provided by the authors are available with the online version of this article. (PDF 515 kb)
